# District health network policy in Iran: the role of ideas, interests, and institutions (3i framework) in a nutshell

**DOI:** 10.1186/s13690-021-00737-7

**Published:** 2021-11-26

**Authors:** Vahid Yazdi-Feyzabadi, Mohammad Bazyar, Sara Ghasemi

**Affiliations:** 1 Health Services Management Research Center, Institute for Futures Studies in Health, Kerman University of Medical Sciences, Kerman, Iran; 2 Department of Health Management and Economics, Faculty of Health, Ilam University of Medical Sciences, Ilam, Iran; 3 Social Determinants of Health Research Center, Institute for Futures Studies in Health, Kerman University of Medical Sciences, Kerman, Iran; 4 Department of Health Management, Policy, and Economics, Faculty of Management and Medical Information Sciences, Kerman University of Medical Sciences, Haft Bagh Highway, Kerman, Iran

**Keywords:** Primary health care (PHC), ‘3i framework’, District health network (DHN), Iran, Case study

## Abstract

**Background:**

District Health Network (DHN), one of Iran’s most successful health reforms, was launched in 1985 to provide primary health care (PHC), in response to health inequities in Iran. The present study aims to use interrelated elements of the 3i framework: ideas (e.g., beliefs and values, culture, knowledge, research evidence and solutions), interests (e.g., civil servants, pressure groups, elected parties, academians and researchers, and policy entrepreneurs), and institutions (e.g., rules, precedents, and organizational, government structures, policy network, and policy legacies) to explain retrospectively how (DHN) policy in Iran, as a developing country, was initiated and formed.

**Methods:**

A historical narrative approach with a case study perspective was employed to focus on the formation and framing process of DHN. For this purpose, the 3i framework was used as a guideline for data analysis. This study mainly searched and extracted secondary sources, including online news, reports, books, dissertations, and published articles in the scientific databases. Primary interviews as a supplementary source were also carried out to meet cross-validation of the data. Data were analyzed using a deductive and inductive approach.

**Results:**

According to the 3i framework, the following factors contributed to the formation of DHN policy in Iran: previous national efforts (for instance Rezaieh plan) and international events aiming to provide public health services for peripheral regions; dominant social discourses and values at the beginning of the Iranian revolution such as addressing the needs of disadvantaged and marginalized groups, which were embedded in the goals of DHN policy aiming to provide basic health services for deprived people especially living in rural and remote areas. Besides, the remarkable social cohesion and solidarity among people reinforced by the Iran-Iraq war were among other factors which contributed to the formation of participatory plans such as DHN (ideas). Main policy entrepreneurs including Minister of Health, his public health deputy and two planners of DHN with similar and rich background in the public health field and sharing the same beliefs (interests) which subsequently led to creation of tight-knit policy community network between them (institutions) also accelerated the creation of DHN in Iran to great extent. Political support of parliamentary representatives (interests), and formal laws such as principles of Iran Constitution (institutions) were also influential in passing the DHN in Iran.

**Conclusions:**

The 3i framework constituents would be insightful in explaining the creation of public health policies. This framework showed that the alignment of laws, structures, and interests of the main actors of the policy with the dominant ideas and beliefs in the society, opened the opportunity to form DHN in Iran.

## Background

The right to health is an essential part of human rights. This issue was first raised in the 1946 constitution of the World Health Organization (WHO) “… the highest attainable standard of health as a fundamental right of every human being.” [1]. In 1948, article 25 of the Universal Declaration of Human Rights introduced health as part of the human right to an adequate standard of living. Achieving equal opportunity in health for all is one of the most popular and pivotal principles entailing to alleviate discrimination and marginalization in access to basic health services [2]. In this regard, global society over the last decades has made efforts to eliminate inequity in health. Accordingly, establishing equity in health within and between countries has always been a global priority [3–5]. In 1978, the inequity in health indicators and distribution of health facilities between and within countries paved the way for the Alma-Ata International Conference in Kazakhstan. This conference placed an urgent action on primary health care (PHC) by the international community to maintain and promote health for all people and improve equity in health [6, 7]. The principles of PHC, proposed by WHO, are social justice and equitable access, appropriate and context-sensitive technology, inter-sectoral collaboration, community participation, and empowerment [8].

### Structure of district health network in Iran

**Fig. 1:**
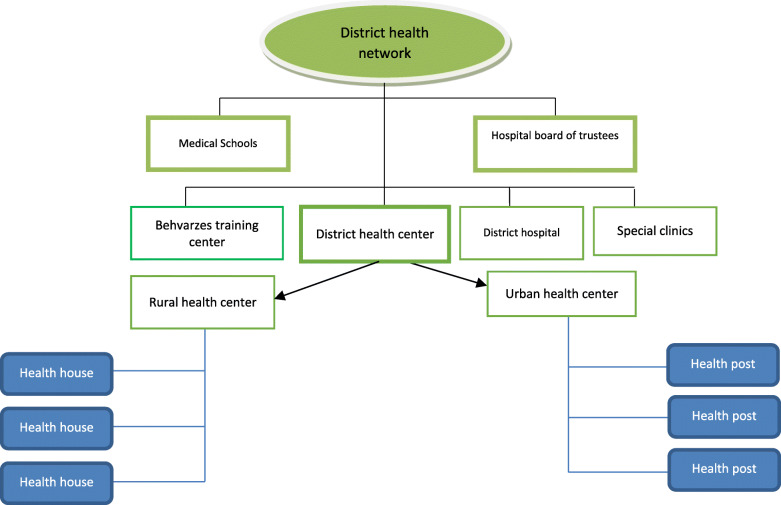
Structure of district heath network in Iran

Iranian healthcare system consists of three main levels of national (Ministry of Health and Medical Education), provincial (universities of medical sciences), and district (health network executive units) [9]. The health network development in Iran occurred in several phases and over the years in urban and rural areas. The first phase (1985) began with establishing health houses (khane-ye-Behdasht) in rural areas. This was the first phase of PHC development in Iran aimed to improve health equity between urban and rural populations [10, 11]. Figure 1 illustrates the structure of current district health network (DHN) in Iran. The main purpose of the structure of DHN is to bring public health services to where people live and work as much as possible. The most peripheral health facilities in rural and remote areas are health houses. They cover approximately 1500 population who live in the main and satellite villages. Each health house is staffed by a female and a male community health worker (Behvarze) [12, 13]. Behvarzes are health forces with basic education who work in the health house located in their own village after being trained to provide primary health care for the villagers. The Behvarzes provide public health care services, including annual census and registration of health information, health education, maternal and child care, family planning, nutritional care and improvement, school health, oral health, immunization, environmental and occupational health occupational health. If a patient needs more professional health services, they are referred by the Bahvarz to upper level, rural health centers (RHCs) which are run by a general physician and health technicians. RHCs monitor and guide activities of the health houses, provide out -patient care as well as refer cases if needed to the district hospitals [13–15].

In general, DHN improved the equity in access to basic health services and facilities to a great extent for rural areas, which has led to significant improvements in health indicators in these regions [16, 17]. The changes of main health indicators over the 30 years (1985–2015) since the policy began have been shown in Table 1.

**Table 1:** The changes of main health indicators over 30 years (1985–2015) since the policy formation of the district health network (DHN) in Iran

Years	1985	1995	2005	2015
Indicators				
**Mortality rate, infant (per 1000 live births)**	56	35.5	21.8	13.8
**Mortality rate, under-5 (per 1000 live births)**	75.1	44.2	25.9	16
**Life expectancy at birth, total (years)**	55.19	68.37	71.91	75.79

Source: The World Bank [18–20].

Understanding how a policy is shaped, what factors influence this formation, how it is implemented, and what challenges it faces make decisions more reliable, realistic, and evidence-informed [21, 22]. However, despite significant advances in health policy analysis in low- and middle-income countries, this domain in these countries is still an underdeveloped issue that needs more attention [23]. Learning from policy reforms can be very informative and helpful for policy makers from other countries, especially in the developing countries facing the challenge of limited resources, to apply them to deal with the same health problems [24, 25].

Moreover, little is known about how the PHC strategy was developed, and the DHN was formed in Iran. It is unclear how actors’ ideas, interests and institutions influenced policy developments and choices. Thus, using the 3i framework (ideas, interests and institutions), the present study aims to retrospectively explain how the DHN policy was initiated and formed in Iran as a developing country. Although the focus of this study is on a PHC in Iran, it might yield important implications for other developing settings that face similar challenges. This issue is important particularly in situations where Iran has historically inspired international society and organizations like WHO to strengthen PHC [26]. Thus, this study may provide some lessons and proper evidence for other health policies in Iran and other developing countries.

## Methods

### Study design

A historical narrative approach with a case study perspective was employed to focus on the formation and framing process of DHN in Iran. The historical approach was used to determine how various policies are connected to changing social, cultural, economic, and political conditions which is common in political science literature and studies [27]. As Berridge (2011) points out, the processes and power relations that have shaped the current policies can be illuminated by historical studies [28]. Knowledge of history is increasingly recommended in health systems research to alert policy formation and formulation to historically affected dynamics [29]. This case study mainly focused on the policy formation of DHN in Iran as a developing country with distinct political, cultural, social and economic conditions to help understanding the specific conditions in which the policy was formed.

### Ideas, interests, and institutions (3i) framework

To show how DHN policy was formed in Iran historically, we applied the 3i framework. This framework explains that the formation of policies and their changes result from the interactions among three factors, including ideas, interests, and institutions, which are described as follows [30].

### Ideas

Idea can include knowledge (scientific evidence and empirical knowledge), values, norms, and beliefs that shape the stakeholders` perspectives [31, 32]. Ideas can affect the way different societal actors define a problem. Ideas can also influence the judgement of policy makers and they may value the effectiveness, feasibility, and acceptabbility of different policy options differently. Ideas can help us understand why different actors such as government, favour certain policy options over others. The values and cultural features dominated in the society at a certain period can clarify why some of the policy options had been put on the agenda [30, 32].

### Interests

Interests defined as the “agendas of societal groups, elected officials, civil servants, researchers, and policy entrepreneurs”. Also, interests describe the powers and preferences of different actors that can change/shape the policies [22, 30].

The conficting interests among different actors and stakehoders, the fact that each actor trying to follow and meet their own interests and the power they have to infuence the process of policy-making to favour their own interest will determine what policy among the possible solutions would be elected. Interests refers to who will be losers or winners by adopting different policies [30, 33].

### Institutions

The element of institutions refers to “collections of structures, rules and standard operating procedures” that shape policy actors’ views, preferences, and behaviors. Others define institutions as “the formal and informal rules, norms, precedents, and organizational factors that structure political behavior” [30, 34, 35].

### Data sources

Several main secondary data sources, including online news media, organizations’ websites, and scientific databases for access to published peer-reviewed articles, books, and dissertations were deeply searched to answer our research questions.

Generally we used three groups of keywords to retrieve relevant documents including:

1. General keywords such as *“history of PHC”, “rural health houses”, “primary health care”, “PHC”, “health network”, “district health network”, “community health worker”, and “behvarz”.* These phrases combined with ‘Iran’ using AND as a boolean Operator.
2. The names of the main founders of the health network in Iran, including Dr. Hossein Malekafzali, Dr. Kamel Shadpour, Dr. Sirus Pileroudi, and Dr. Alireza Marandi were searched
3. Contextual keywords such as “Social context”, “Political context”, and “cultural context”. These phrases combined with ‘Iran’ and “1980s” to understand the context in which the DHN policy was formed.

The search operation was performed to retrieve scientific articles from international databases including Web of Science, PubMed, and Scopus. Iranian databases such as Noormags, SID, Barakat knowledge network system, Magiran were searched as well. The exact search strategy considered appropriate to the searched databases. Articles were searched in Persian and English languages. Out of 1071 retrieved articles, 520 articles were identified as irrelevant by reviewing the titles. After reviewing the full text of the articles, some articles were deleted because they were irrelevant to the subject. Finally, 30 articles were included in the study.

The retrieved documents were then screened using four criteria, including authenticity (being original and genuine), credibility (accuracy), representativeness (being representative of the totality of the documents in their class), and meaning (what they say). We also manually searched official documents of historical events and compiled policy documents produced by founders of the DHN and the Ministry of Health and Medical Education (MoHME). These documents mainly explained the macro-economic and socio-political contexts, and the role of particular actors and organizations in the formation of DHN in Iran. The published memoirs of key actors were collected and retrieved from online news media, organizations’ websites, and books. Several general and specialized online news media in Iran including Mehrnews, Farsnews, Webda, and News media related to the Iran medical universities were also visited. We also contacted the main authors who worked on the PHC in Iran to find more relevant documents.

One of the obstacles of historical research is that historical documents regarding the policy might have not been recorded wholly or in part. We tried to fill this gap as much as possible by doing interviews with those who had engaged in the process of passing the DHN policy in Iran or had knowledge about it. Although we mainly relied on secondary sources, we interviewed some of key informants, including one of the former health ministers, two official authorities from MoHME, and three  Academicians who were knowledgeable about formation of DHN**.** We triangulated these interviews with secondary sources.

Table 2 demonstrates the type and number of each source:

**Table 2:** The type and number of each source used in this study regarding the history of formation of district health network in Iran

References		Descriptions	Number of included records
Documents (Doc)	*Books (Doc1)*	• Books on the establishment of DHN in Iran • Books on the memories of the main actors about establishment of DHN	6
	*Published Articles (Doc2)*	• Articles on the establishment of District Health Network in Iran • Articles on the international conditions and PHC policy • Social and political context of Iran at the time of DHN formation	30
	*Presentations and Reports (Doc3)*	• Documents and reports containing published interviews with the main actors • International reports on the PHC strategy • National reports and presentations on the DHN formation	20
	*Dissertation*	• A Ph.D thesis about the challenges ahead of development of PHC network in Iran health system	1
Online News Media (N)	*The Official News Agency of the Ministry of Health (Vebda)*	Vebda news agency is affiliated with the Iran’s Ministry of Health and Medical Education and publishes only health news.	25
	*News Agencies Affiliated to the Medical Universities in Iran*		20
	*Mehr News Agency*	These news agencies report all daily news, which only health-related news sites were searched in this study.	60
	*Fars News Agency*		
	*Isna News Agency*		
Interviews (I)	*Former Health Minister*	Dr Marandi who was the health minister at the time of launching the Health Network in Iran	1
	*Official Authorities*	Official Authorities who have relatively rich experience in the public health field	2
	*Academicians *	Academicians who had rich information about formation of DHN	3

### Data analysis

A hybrid approach of inductive and deductive thematic analysis was employed within the paradigm of interpretivism. All non-text materials were transcribed and summarized verbatim. All of them were read several times, and then all transcribed texts were coded one by one using inductive data-driven open coding. In this stage, the data’s patterns and associations were searched, and all new inductive codes were assigned to concepts that emerged from the data. The findings were then subjected to deductive analysis using the 3i framework. We also used inductive approach to accept new concepts emerging from the data analysis. This process was ongoing, organic, and iterative.

### Trustworthiness of results

At all stages of qualitative study, credibility, transferability, dependability, and confirmability criteria were employed [36]. The triangulation of data sources technique help to ensure credibility [37]. For the purpose of transferability, we tried to describe the context of the country and the organizational structure of the Ministry of Health. Furthermore, the details of research questions, data gathering, screening, and data analysis were described. To ensure dependability, one of the authors (MB) acted as an external auditor and reviewed the findings to evaluate whether the data supported the findings, interpretations, and conclusions. All perspectives related to the formation of the PHC setting were welcomed and tried to keep away bias from one specific belief about research to enhance the confirmability.

## Results

The present study’s findings are categorized into three main themes according to the 3i framework’ components: ideas, interests, and institutions.

### Ideas

In the ideas section, two main points are presented: evidence and experiences related to the history of DHN in Iran; and dominant values and beliefs in the society.

#### Evidence and experiences related to the DHN in Iran

The ​​DHN policy in Iran, in response to the global approach of “Health for All by 2000”, was launched in 1985 [38]. However, ideas do not originate from a single source, and it would be necessary to pay more attention to different aspects of an idea and go beyond the “usual suspects” [39].

At the national level, different documents and health indicators showed a great difference between the health status of the urban and rural areas in Iran in the 1960–70 decade [40]. Regarding these disparities, the law of Health Corps was proposed as one of the national reform principles [41]. In the 70s and in line with global trends, Rezaieh plan in partnership with WHO was proposed and implemented, aiming to find a strategy to use non-physician health workers to provide healthcare services for people living in rural and hard to reach areas:

*"A village in Rezaieh (currently Urmia City in northwestern of Iran) was selected as the pilot place for this project … In fact, this project was implemented with the collaboration of the Ministry of Health, the School of Public Health of Tehran University of Medical Sciences, and the WHO, in 17 villages of West Azerbaijan Province in 1972. The plan was very successful in Azarbaijan but failed at the stage of national development"* (Policymaker (Doc3) Health Development in Iran Ministry of Health).

Similar plans before the Rezaieh plan were implemented in Iran with different degrees of success. In 1940, the assistant physicians’ (behdār) training plan in Iran was initiated, which stopped in the 1960 decade. Other initiatives such as the Selseleh (Alashtar) project, and the project of training assistant physicians (behdār) in Kavar, Fars province, were also implemented but failed to develop a sustainable strategy for bringing basic public health services into remote areas. These efforts, especially the Rezaieh plan and chronic disparities in health status between the urban and rural areas, led to formation of the idea of “District Health Network” in Iran.

At the international level, events and evidence paved the way for introducing of primary health care in the world. The following events, were among the main international factors which supported the idea of establishing DHN in Iran: *Kennedy’s Peace Corps in 1961 (which was implemented later in Iran in the form of Literacy Corps and Health Corps);* China’s barefoot doctors in 1970 (rural workers’ training for basic disease-prevention); election of Dr. Mahler as the WHO’s general director in 1973 who greatly insisted on social justice in global health; and Alma-Ata Declaration in 1978 [42–44].

#### Values, beliefs and discourses

Policy-making is also affected by dominant values, beliefs, and discourses in society [31]. In 1979, the Iranian Islamic revolution happened. One of the main discourses of this revolution was to improve living conditions and equity for disadvantaged groups and combating against poverty and deprivation [45]. Fortunately, the goal of the DHN policy was in line with the mission of revolution of Iran. This policy aimed to increase access to health facilities and improve equity in health for deprived people in rural areas. This facilitated the acceptance of the DHN policy in those years.

*“We were supposed to help disadvantaged groups, especially rural residents, and we thought this was our responsibility toward these people"(*Former Health Minister (I))*.*

On the other hand, Iran was at war. The Iran-Iraq war began in 1980. This crisis boosted social cohesion and solidarity among Iranian people [46, 47]. This led to a supportive context for launching participatory plans such as DHN:

*"The participation of people who had strong motivations and wanted to help the country would provide a good opportunity for improving the services. Iran was at war. Everybody had the zest of the revolution, and it was a very good time to work"* (Politician in Health System- (N) Tehran University of Medical Sciences News).

*“Although the war weakened the country's infrastructure, it also strengthened the spirit of solidarity, and everyone was sympathetically seeking to improve the condition”* (Official Authorities (I)).

Table 3 demonstrates the most significant events related to the formation of PHC globally and DHN in Iran, in summary.

**Table 3:** Most significant events related to the primary health care in the world and District Health Network in Iran

Dimensions of the Framework	Issues related to the establishment of health houses in Iran
Ideas	**Trends, experiences and evidence related to the idea of “Health Network Policy” in Iran:** **-International** • Establishment of the World Health Organization; Kennedy’s Peace Corps Thesis, The China’s barefoot doctors, strengthening of Social Justice Approaches in Global Health; Alma-Ata International Conference. **-National** • Training of Assistant Physicians (Behdār) in Iran, the establishment of Health Corps, Training of Assistant Physicians (Behdār) in Kavar (Fars Province), Rezaieh National Project. **-Beliefs and discourses:** Dominant discourse: helping the disadvantage groups especially rural area Empathy and social cohesion: supportive context for participatory plans
Interests	• Existence of opposition to DHN policy and overcoming it by advocacies and almost aligning the interests of the parliament with the goals of DHN policy
Institutions	• **Rules:** Article 20, 29 and 43 of the Iranian Constitution • Addressing the health and sanitation status of rural areas was one of the main expectations of people from the government in that time • **Policy Network:** Establishment of the network with strong relationships between designers of DHN policy and politicians (Minister of health and his deputy) due to the old friendship and common experiences and beliefs.

### Interests (actors)

The policy-making process moves forward through networks of stakeholders with their interests and motives [48]. Different individuals, groups, and organizations were interested in the formation of the DHN policy in Iran. However, three groups were among the main stakeholders. These groups were classified into internal (inside the health sector) and external (outside of the health sector) stakeholders. The first group was the rural dwellers. At that time, about half of the population was living in rural areas [49]. Rural dwellers benefited from the implementation of DHN as it improved health facilities in rural areas. At the same time they had an active and strong role in implementation of the policy. High participation and acceptability of DHN policy by the rural people shows that they supported and welcomed the implementation of the policy. The next influential group was the key founders of DHN. These were Dr. Pileroudi and Dr. Shadpour as the main designers of the DHN program and Dr. Marandi, the Minister of Health (from 1984 August 18 to 1989 August 29) and Dr. Malek Afzali as his public health deputy. They were the policy entrepreneurs and main actors in putting the DHN policy on the agenda. They had the same working experience in rural areas and were quite familiar with the common rural health problems [10, 50, 51].

The third group were those who supported the policy indirectly, such as representatives that approved the bill in the Parliament. However, there were groups from the ministry of health who were against the DHN policy. Some of these opposing actors were physicians who preferred treatment and hospital-based services over public health and preventive medicine and considered having physicians in rural areas more equitable and effective than having health workers [50, 52, 53].

*“… .The first slide that Dr. Manafi (former minister of health before Dr. Marandi) put on the screen explained that they wanted to build a health house. The Members of the Parliament said, 'We want a thousand-bed hospital, but you want to build a health house?!' Health houses served many people, but there was no understanding of the real value of a health house at that time”* (Politician in the health system (N), ISNA News).

*"It was the first years of the revolution, and we were strongly accused that we were going to waste time. In the absence of the country's appropriate economic and social infrastructures, we were not supposed to talk about health and provide a solution"* (Policy-maker (Doc1) District Health Networks, Pileroudi).

Also, some officials believed that the main founders of DHN, Dr. Kamel Shadpour and Dr. Sirus Pileroudi, belonged to the former regime [51].

As a result, at the beginning of the revolution, the above policymakers (Dr. Shadpour and Dr. Pileroudi) were isolated [51]. Despite these political oppositions, eventually, the DHN policy was supported and accepted at that time. Democracy and supporting the oppressed was the motto of most groups. It should be noted that there were ‘left and right’ parties with different views about the appropriate mechanisms to realize the social justice. The votes from the second parliamentary elections - coinciding with the approval of the DHN - showed that the left-wing had occupied more seats in the parliament than the right-wing. The left-wing believed that the government should play a key role in establishing social justice, especially through investment in economic development [52]. Thus, this situation supported the idea of establishing DHN policy developed by the policymakers and subsequently approved the DHN policy based on justice-oriented and government budget. In fact, from the policymakers’ perspective, applying the DHN policy might help to improve the health of people with low socio-economic status [53].

### Institutions

Long-standing friendships among Dr. Shadpour and Dr. Pileroudi, and the Public Health Deputy of MoHME (Dr. Malekafzali) created a strong *policy network* between them. The power of this network, and the advocacy of its memebers for the public health approach opened the path for getting the “DHN” policy on political agenda. Old friendship, common values, preferences, and experiences (working in Health Corps in the deprived rural areas and the Rezaieh plan) between the main members of the policy network created a desire for DHN formation policy. Also, the appointment of Dr. Marandi as the minister of health increased the authority to apply this idea [10, 50–53].

Although the adoption and formation of a policy are largely dependent on the interests of actors and their behavior [54], the capacity of political actors to promote a policy successfully can be undermined, in some specific circumstances, such as the lack of resources or strong organizational barriers [22]. In the early years after the revolution and during the Iran-Iraq war, Iran’s economic infrastructure was damaged, and the formation of a national health plan was hampered.

Articles 20, 29, and 43 of the Iranian Constitution, address the non-discrimination between sections of the society,  the right to health for all, and the eradication of poverty and deprivation respectively [55]. These articles were the main formal laws which supported the passing the law of DHN in Iran. 

In addition to formal laws, considering the motto of the Iranian revolution, i.e., “supporting the poor”, Iranian people expected the government to address the health and sanitation status of the rural areas:

*“It was a revolution; people welcomed the revolution hoping for better conditions. It was their inalienable right; we were responsible for these people”* (Former Health Minister (I)).

## Discussion

**Fig. 2:**
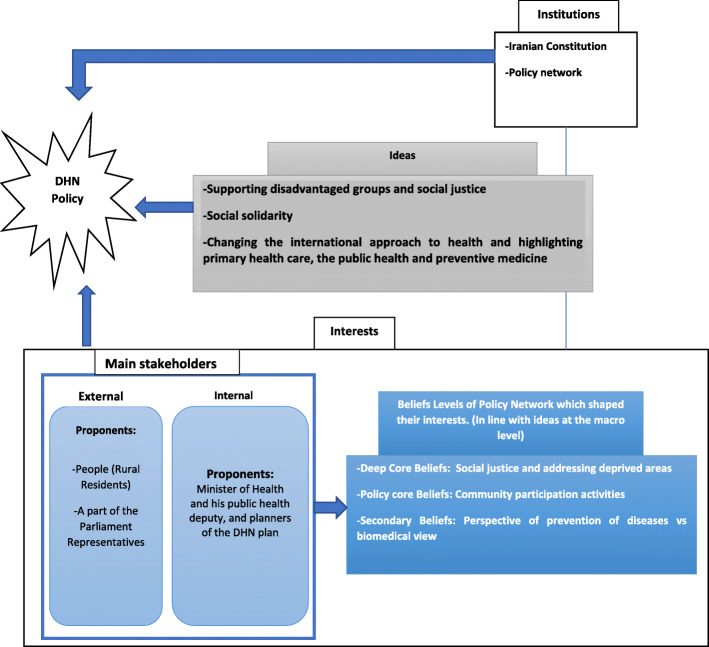
Interactions between three components of 3i framework and the policy network regarding the formation of DHN policy in Iran

Learning from history is one of the pieces of evidence that is used for sound policymaking. Lessons learned from the past create added value for policymaking [56]. Studying how policies have been successfully made in the past can improve the future policy-making process. The present study aimed to explain how the ‘District Health Network’ policy was made in Iran based on the three components of the 3i framework. The results showed that ideas with the concept of values, beliefs, and discourses greatly impacted the formation of this policy. Such ideas can shape the behavior of policymakers and politicians [57]. Also, Kingdon argued that ideas, especially those related to people’s worldviews, play a major role in motivating them to pursue their interests [39]. Figure 2 demonstrates the interaction between ideas, interests and institutions, and the policy network concerning the formation of health network policy in Iran.

However, this does not mean that ideas play a great role in shaping a policy in all circumstances. It is important to note that the importance of ideas, interests, and institutions in the policy-making process depending on the policy [58]. Mark Blyth stated that ideas might play a more prominent role in shaping actors’ interests and, ultimately, a policy, especially in uncertain circumstances such as war or economic crisis [59]. When the DHN policy was proposed in Iran after the revolution, while Iran was at war with Iraq and economic infrastructure had been damaged severely, the ideas probably played a greater role in shaping the actors’ interests.

The actors’ behaviors and actions to achieve their interests can play a decisive role in shaping a policy. These actions and behaviors, particularly, when create a network or coalition among the main actors, can have great power. The ‘survival of mothers’ in Tanzania is an example put on the agenda just after creating of political coalition among actors, which increased their political power [60, 61]. Regarding the (DHN) policy in Iran, the relations between the main actors based on old friendships and common beliefs led to forming a strong network to put the health network policy on the agenda. Sharing knowledge, experience, and political power by the network members, i.e., the Minister of Health and his public health deputy, and influential organizational positions in the Ministry of Health paved the way for approving and putting this policy on the agenda.

The formation of networks is dependent on how the beliefs join each other. Regarding the ‘DHN policy’ in Iran, a policy network was formed between the policymakers and the prominent politicians. Investigating individuals’ opinions and beliefs in this network showed that they had similarities in three layers of belief (deep core beliefs, policy core beliefs, and secondary beliefs). Sabatier and Jenkins-Smith formulated this three-tiered hierarchical belief system of actors in 1999 in the Advocacy Coalition Framework (ACF) theory, a theory for understanding policy process and its changes. The highest layer is named deep core beliefs. It includes fundamental beliefs with very resistance to change. In contrast, policy core beliefs as the second layer include empirical beliefs covering an entire policy subsystem with less resistance to change. They consist of strategies for achieving normative principles of deep core beliefs. Finally, the secondary beliefs include policy preferences which are related to a subcomponent of a policy subsystem. This layer may include preferences of policy participants/actors or specific government tools for achieving objectives of a given policy [62–64]. It should be noted that there were shared values and beliefs in three layers among the main actors of the DHN. As social justice and eliminating health inequalities played as- deep core beliefs. Furthermore, the agreement among main actors to use of community-based and participatory approach and plans -, and a shared aggrement on disease prevention strategies instead of concentrating on hospital-based services among main actors composed the policy core beliefs and secondary beleifs respectively .

In his study, Schmidt suggested that ideas and discourses are important when influencing the actors’ interests and overcoming institutional barriers [65]. Institutional barriers existing at that time in Iran, just like damaged economic structures and some conflicts of interests, hindered DHN policy formation to some extent. However, the DHN policy compliance with the prevailing ideas and discourses overcame the barriers. In fact, by raising the problem of differences in health indicators between urban and rural areas and proposing extending PHC services to rural and deprived areas, the idea of DHN policy consistent with prevailing social values such as supporting the underprivileged populations could overcome institutional barriers and conflicts of interests.

Moreover, ideas with the concept of knowledge, evidence, and experiences are also presented as policy solutions [66]. The movement of policy solutions worldwide towards diseases-prevention instead of the biomedical approach, the use of indigenous forces to promote health, and the successful results of the Rezaieh and other similar plans in Iran also had a major impact on developing a DHN in Iran.

Ideas not only affect institutional barriers but are sometimes converted into specific rules themselves [67]. Regarding the health network in Iran, some institutional laws that helped this policy were in line with existing ideas and discourses. For example articles 20, 29 and 43 of the Iranian Constitution were among these laws [55].

### Study limitations

This study had some limitations. The generalizability of results might be a source of limitation for this study, as a case study approach was used to explain aspects of formation and development of a specific policy named (DHN) in Iran. However, the theoretical paradigm of constructivism allows us to focus on a single policy to explain how to form a policy and provide a thick description to enhance transferability of results to other settings in Iran or developing countries. Another limitation of the present study is that analyzing the historical processes depends on data availability and historical archives, which is not always met. Thus, considering the restricted access to oral history, we inevitably build our analysis on secondary sources and official publications, which restricted our ability to detect some key individuals and organizations. There are details of what was historically occurred, especially in the early Islamic revolution of Iran, which cannot be well seized within this article’s space. Last but not least, on an aggregate basis, we had to restrict our study to the most common and accessible instances of the case studied.

## Conclusions

Regarding the formation of the DHN policy in Iran, the role of ideas was more prominent than interests and institutions. Also, findings showed that the alignment of laws, structures, and interests of the main actors of the policy with the dominant ideas and beliefs in the society, opened the opportunity to form DHN in Iran. In general, the policymaker’s awareness of the prevailing ideas and their impact on the actors, general population, and even the policymakers’ thoughts help to facilitate the approval of a policy.

## Data Availability

The corresponding author will gladly provide any supporting materials upon request.

## Abbreviations

DHN: District health network
PHC: Primary health care
WHO: World Health Organization
MoHME: Ministry of health and medical education
3i framework: Ideas, interests, and institutions framework
